# Hepatocellular carcinoma: HCC

**Published:** 2011-02-01

**Authors:** Hubert E. Blum

**Affiliations:** 1 Department of Medicine II, University of Freiburg, Freiburg, Germany

**Keywords:** Hepatocellular carcinoma

Hepatocellular carcinoma (HCC) is one of the most common malignant tumors worldwide. The annual incidence ranges from <10 cases per 100,000 persons in North America and western Europe to 50-150 cases per 100,000 persons in parts of Africa and Asia, where HCC is responsible for a large proportion of cancer-related deaths. However, a rise in the incidence of and mortality from HCC, most likely reflecting the increased prevalence of hepatitis C virus (HCV) infection, obesity, and diabetes mellitus, has been observed in most industrialized countries [1][2][3][4].

The major etiologies of HCC are well defined including: Chronic viral hepatitis B, C, and D; Toxins and drugs (e.g, alcohol, aflatoxins, anabolic steroids); Metabolic liver diseases (e.g., hereditary hemochromatosis, alpha-1-antitrypsin deficiency); Diabetes mellitus, obesity in men, nonalcoholic fatty liver disease (NAFLD). Some of the steps in the molecular pathogenesis of HCC have been elucidated in recent years. Little is known about the incidence and causes of HCC in Iran-particularly in the large province of Kerman, in southeast Iran, which has a population of approximately 2.6 million people.

In recently published article by Hepatitis Monthly, Dr. Sodaif Darvishmoghadam and colleagues, presented a detailed analysis of the epidemiology of HCC in this province and compare it with nationwide data from a report by the Iranian Cancer Registry Program, published in 2008 [5][6]. Between 1999 and 2006, 95 cases of confirmed HCC were reported in Kerman, corresponding to a crude annual incidence of 0.522 cases per 100,000 persons. The standardized annual incidence was 0.7 per 100,000 persons. Nationwide, the crude and standardized annual HCC incidence was 0.199 and 0.2 per 100,000 persons, respectively. In males, it was 0.9 and 0.4 in females, indicating an approximately 2-fold higher risk for HCC in men compared with females. Further, the authors demonstrated that HCC patients in Kerman are significanty younger than in Iran in general. The incidence of HCC is significantly lower in Iran and its provinces compared with other parts of the world, including Africa, East Asia, North America, Western Europe, and the Middle East, presumably due to the low incidence of hepatitis B virus (HBV) and HCV infection as well as alcoholic liver disease. Notably, in Iran there are provinces that experience an extremely low number of HCC cases annually, such as Ardebil, Guilan, and Golestan, and provinces that have a low but significantly higher incidence, such as Kerman, Fars, Razavi Khorasan, and most notably Tehran, Kerman province showing the highest crude annual incidence. The reasons for these geographic differences in the mean number of annual HCC cases, the crude annual incidence, and the distribution of ages in patients with HCC in Iran have not been determined. Further studies should address these issues to reduce the incidence of HCC. In this context, in addition to risk factors, preventive compounds, such as coffein [7][8][9], should be considered. Such studies are particulary important for provinces in Iran that have a higher incidence of HCC, where it remains a devastating malignant disease with very limited therapeutic options.
